# Evaluation of macro- and meso-mechanical properties of concrete under the aggressiveness of landfill leachate

**DOI:** 10.1038/s41598-022-07418-7

**Published:** 2022-03-10

**Authors:** Wei Feng

**Affiliations:** Institute of Architecture and Civil Engineering, Yulin University, Chongwen West Road 4, Yulin, 719000 China

**Keywords:** Chemical engineering, Civil engineering

## Abstract

Current researches on the mechanical properties of concrete are mainly concentrated on the aggressiveness of sulfate, chloride ion, acid and freeze–thaw cycles. However, the evolution of concrete mechanical properties aggressived by landfill leachate remains to be revealed. As the micro-nano indentation test is a new method to measure the fine/micro mechanical behavior of materials, it is used in this research to evaluate the micro-mechanics of the landfill leachate of the concrete sample surface and different aggressived depths. In addition, the evolution of the macro-mechanical properties of concrete aggressived by the landfill leachate was studied through uniaxial compression tests. The results show that the hydration products with high elastic modulus are consumed by the aggressiveness of landfill leachate, which results in a large number of microscopic cracks on the surface of the concrete. Moreover, the longer the aggressive time, the shallower the aggressive depth, and the more severe the deterioration of the micromechanical properties of the sample surface. It is also notable that the uniaxial compressive strength of the concrete samples aggressived by landfill leachate showed a linear increase and then gradually decrease. Comparing with the sodium chloride and sodium sulfate solutions, the landfill leachate has the most significant weakening effect on the elastic modulus of concrete.

## Introduction

Generally, the mechanical properties of concrete behave a positive correlation with the strength of its anti-aggressived ability. The better the mechanical properties, the stronger the ability of resisting aggressive ions, and the harder the internal structure to be destroyed^1,2^. So far, very few researches have been concentrated on the mechanical properties of concrete after being aggressived by the landfill leachate, and most researches have been focused on the study of the mechanical properties of concrete aggressived by the sulfate^3–6^, chloride ion^7,8^ and freeze–thaw cycles^9,10^. However, researches on the evolving of the mechanical properties of concrete aggressived by landfill leachate remains to be done.

Generally, concrete aggressiveness is a process evolves from surface to inside, which can be divided into aggressived area and non-aggressived area. Since aggressived depth is one of the important parameters to distinguish the aggressived and non-aggressived areas, and traditional methods measuring concrete aggressived depth includes chemical analysis, ultrasonic detection and strength degradation, which have a certain degree of deficiencies. For example, the chemical analysis method has high accuracy, but it is not suitable for the detection of a variety of aggressive ions^11^. Even though the ultrasonic detection method has poor accuracy, it is suitable for determining sample with large aggressived depth^12,13^. And the strength degradation method assumes that the aggressived area does not bear force, which is inevitably inconsistent with its actual features.

Past researches took the aggressived and unaggressived area of concrete as a whole to evaluate its mechanical properties, and then determine the degree of deterioration and establish the constitutive relationship of the aggressived concrete^14,15^. Actually, the mechanical properties at different aggressived depths are different, and the test results are greatly affected by the sample size and degree of aggressiveness. Therefore, it is necessary to study the mechanical properties of concrete at different aggressived depths.

Recently, a new method of micro-nano indentation test was invented to measure the fine/micro mechanical behavior of materials, which has been widely used in the metal materials and cement-based materials^16^. The emerging nanoindentation technology effectively solves the defect that the traditional indentation measuring method is only suitable for larger size sample. The continuous changing of the load is controlled by the computer, and the indentation depth is measured in real time. Because of the ultra-low load characteristic, the monitoring sensor has a displacement resolution smaller than 1 nm. Therefore, it can detect a pressure depth as small as nanometers (0.1–100 nm), which is especially suitable for measuring the mechanical properties of ultra-thin materials such as thin films and coatings, as well as measuring mechanical properties of a material on the nanometer scale^17^.

Therefore, the micron indentation test was conducted to study the micro-mechanical properties of the concrete sample surface and different aggressived depths for different time by high-concentration landfill leachate aggressiveness group HLLN (High concentration—Landfill leachate—No osmotic pressure), landfill leachate original solution aggressiveness group NLLN (Normal concentration—Landfill leachate—No osmotic pressure), landfill leachate original solution plus osmotic pressure aggressiveness group NLLO (Normal concentration—Landfill leachate—Osmotic pressure). At the same time, the uniaxial compression test is also used to study the evolution of macro-mechanical properties of the concrete suffering aggressiveness of high-concentration landfill leachate group HLLN, sodium sulfate solution group NSSN (Normal concentration—Sodium sulfate solution—No osmotic pressure) and sodium chloride solution group NSCN (Normal concentration—Sodium chloride solution—No osmotic pressure). Moreover, related test results can be used for providing scientific basis for understanding the evolution of macro/micro mechanical properties of concrete structures under the aggressiveness of landfill leachate.

## Aggressiveness tests

### Test materials and aggressiveness method

Because of the limited aggressiveness rate of the landfill leachate on concrete under natural conditions, the concentration of sulfate ion and chloride ion were increased in the landfill leachate to accelerate the aggressiveness effect^18^. At the same time, considering the differences in aggressiveness environments, five groups including high-concentration landfill leachate aggressiveness group HLLN (High concentration—Landfill leachate—No osmotic pressure), sodium sulfate solution aggressiveness group NSSN (Normal concentration—Sodium sulfate solution—No osmotic pressure), sodium chloride solution aggressiveness group NSCN (Normal concentration—Sodium chloride solution—No osmotic pressure), landfill leachate original solution aggressiveness group NLLN (Normal concentration—Landfill leachate—No osmotic pressure), and landfill leachate original solution plus osmotic pressure aggressiveness group NLLO (Normal concentration—Landfill leachate—Osmotic pressure) were prepared. Since the contents of sulfate ion and chloride ion in the landfill leachate are relatively high^18^ and has greater impact on the concrete, the total concentrations of sulfate ion and chloride ion in group HLLN were increased to 50 g/L, respectively, which can accelerate the aggressiveness effect. The concentration of the remaining components was the same as that in group NLLN. In order to study the difference of aggressiveness conditions under high-concentration landfill leachate and single sodium sulfate plus sodium chloride solution, the concentrations of sulfate ion and chloride ion in group NSSN and group NSCN were set 50 g/L.

The indoor configuration of landfill leachate was used in this study to ensure the unity of the experimental variables, which can effectively avoid the influence of the composition of landfill leachate on the experimental results. The composition ratio of landfill leachate was selected according to^19^, and the composition ratio of each group of solutions are shown in Table 1.

**Table 1:** Composition ratio of landfill leachate.

Component	HLLN	NSSN	NSCN	NLLN	NLLO
Acetic acid (ml/L)	3.5	0.0	0.0	3.5	3.5
Propionic acid (ml/L)	3.5	0.0	0.0	3.5	3.5
Dipotassium hydrogen phosphate (mg/L)	15.0	0.0	0.0	15.0	15.0
Potassium bicarbonate (mg/L)	156.0	0.0	0.0	156.0	156.0
Potassium carbonate (mg/L)	162.0	0.0	0.0	162.0	162.0
Sodium chloride (mg/L)	47,588	0.0	50,000	720	720
Sodium nitrate (mg/L)	25	0	0	25	25
Sodium bicarbonate (mg/L)	1506	0	0	1506	1506
Calcium chloride (mg/L)	1441	0	0	1441	1441
Magnesium chloride hexahydrate (mg/L)	1557	0	0	1557	1557
Magnesium sulfate (mg/L)	78	0	0	78	78
Ammonium bicarbonate (mg/L)	1220	0	0	1220	1220
Ferrous sulfate (mg/L)	500	0	0	500	500
Copper sulfate pentahydrate (mg/L)	20	0	0	20	20
Manganese sulfate (mg/L)	250	0	0	250	250
Sodium sulfate (mg/L)	49,194	50,000	0	0	0

As the aggressiveness effect of the landfill leachate develops, the hydration products in the concrete reaction with the harmful substances in the landfill leachate, which inevitably influence the concrete durability. Aggressive ions such as sulfate ions and chloride ions in the landfill leachate will chemically reaction with the C–S–H gel and calcium hydroxide in the concrete hydration products, thereby causing aggressiveness to the concrete^1–8,18,20^. In addition, the ordinary silicate cement and primary fly ash were used for the tests, and the fine aggregate was medium sand with a fineness modulus of 2.6, and the coarse aggregate was 5–25 mm continuously graded gravel. The chemical composition of cement and fly ash can be seen in Table 2. The concrete mix ratio is selected with cement:water:fly ash:fine aggregate:coarse aggregate = 1.00:0.59:0.25:2.76:3.79.

**Table 2:** Chemical compositions of cementitious materials (%).

Chemical compositions	SiO_2_	Al_2_O_3_	Fe_2_O_3_	CaO	MgO	SO_3_	K_2_O
Cement	21.47	5.80	4.04	56.64	3.24	2.08	0.22
Fly ash	52.54	31.22	5.61	4.615	0.64	1.208	1.337

**Figure 1:**
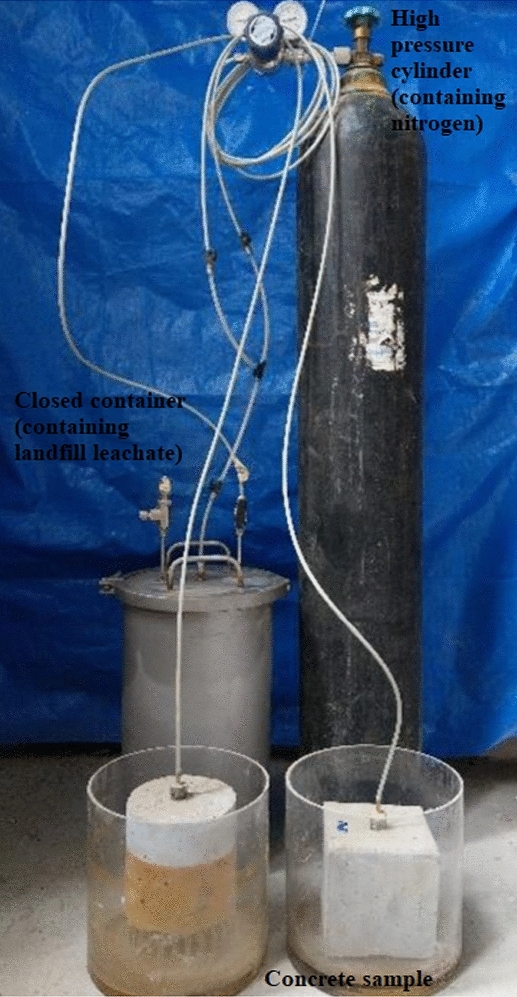
Osmotic pressure device.

The cylindrical specimens (φ150 mm × 150 mm) were cured under standard curing conditions (Temperature 20 ± 2 °C, humidity 95%). After the maintenance was completed, the standard cylindrical specimen of φ 50 mm × 100 mm is prepared by coring, cutting and grinding. And then they were put in the high-concentration landfill leachate aggressiveness group HLLN and landfill leachate original solution aggressiveness group NSSN, respectively, so as to simulate the aggressiveness of concrete structure under different environments. Considering the underground concrete structure on the landfill site is occasionally aggressived by the flowing landfill leachate with certain pressure, the high-concentration landfill leachate aggressiveness group HLN, sodium sulfate solution aggressiveness group NSSN, sodium chloride solution aggressiveness group NSCN, landfill leachate original solution aggressiveness group NLLN, and landfill leachate original solution plus osmotic pressure (apparatus shown in Fig. 1) aggressiveness group NLLO were prepared, among which aggressiveness groups HLLN, NSSN, NSCN, NLLN were used for conventional immersion aggressiveness without osmotic pressure, and the group NLLO was used for immersion conventional immersion aggressiveness with osmotic pressure. And test samples for the group NLLO were the cylindrical samples of φ150 mm × 150 mm. In addition, a hole with a diameter of 20 mm and depth of 100 mm was drilled in the center of the cylindrical sample. As shown in Fig. 1, a high-pressure gas cylinder is used to pass nitrogen into a closed container containing waste leachate, thus providing osmotic pressure to the waste leachate. Under the osmotic pressure, the waste leachate will flow into the cylindrical hole inside the cylindrical specimen, so that the waste leachate under pressurized condition can erode from the inside to the outside of the concrete.

### Micron indentation tests and principles

In order to obtain the evolution law of the meso-mechanical properties of concrete aggressived by landfill leachate, specimens aggressived by the high-concentration landfill leachate aggressiveness group HLLN, original concentration of landfill leachate aggressiveness group NLLN, and original solution of landfill leachate plus osmotic pressure aggressiveness group NLLO were tested through micron indentation tests.

Since the concrete sample is a three-phase structure composed of hydration products, fine aggregates and coarse aggregates, and the aggressive target of the landfill leachate on the concrete is hydration products, aggressiveness on the fine aggregates and coarse aggregates are ignored in this study. According to the loading indentation tests about unaggressived specimens under different loads (5, 15, 30, and 50 N), it is concluded that no deformation damage occurred on the surface of the indentation point when the concrete suffered a maximum load of 15 N, whose indentation depth was 35 μm and indentation diameter was 0.25 mm. When the load is small (5 N), the indentation depth is shallow and generally affected by the surface roughness. However, large loads (30 N, 50 N) led to the collapse of the surface of indentation point, which eventually caused errors in the measurement results.

**Figure 2:**
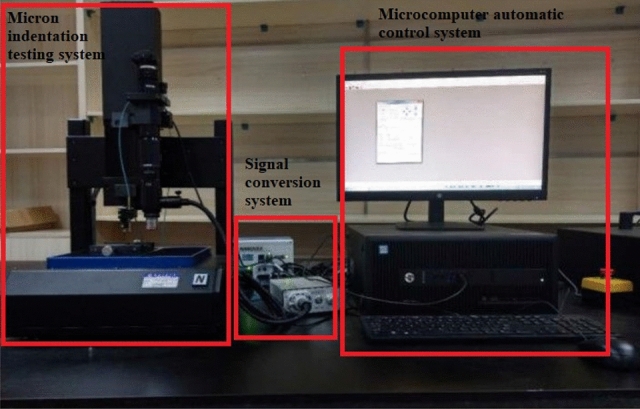
Micron indentation testing system.

According to the test results and standard^21^, the maximum force of 15 N was determined in the indentation tests, and the distances from the indentation center to the boundary of the cubic sample and coarse aggregate are 2 mm, respectively. The test temperature was maintained at 24 ± 2 °C, and the micro-indentation test system is shown in Fig. 2. The distance between the centers of the two indentations are also 2 mm. In addition, a load control mode was used in the whole tests. Firstly, the sample surface was loaded linearly to 15 N with a speed of 30 N/min, and the constant load was kept constant for 10 s. Finally, samples were linearly unloaded with a speed of 30 N/min. Hence, the indentation depth curve can be obtained and the modulus of the elasticity and hardness of the hydration products can be calculated. Based on the above principles, the micron indentation tests were conducted on the cubic samples and cylindrical samples (three samples were selected in each group, each sample subjected to 30 times indentation tests).

**Figure 3:**
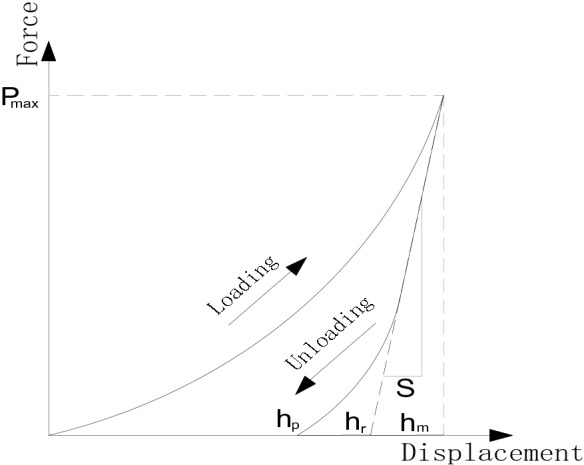
Loading/unloading-displacement curve.

Figure 3 shows a typical load/unload-displacement curve. The horizontal axis *h* is the indentation depth of the indenter, vertical axis *P* denotes the load, *h*_*m*_ means the maximum indentation depth during tests, *h*_*p*_ indicates the depth of the residual indentation after unloading, *h*_*r*_ represents the intersection between the tangent line and the horizontal axis at the maximum load of the unloading curve, and *P*_*max*_ is the maximum testing load. Based on the Oliver-Pharry principle^22^, the elastic modulus *E* of a material can be calculated according the following formula:

1 $$\frac{1}{{E_{r} }} = \frac{{1 - v^{2} }}{E} + \frac{{1 - v_{i}^{2} }}{{E_{i} }}$$

2 $$E_{r} = \frac{\sqrt \pi }{{2\beta }}\frac{S}{{\sqrt {A(h_{c} )} }}$$

3 $$S = \left( {\frac{dP}{{dh}}} \right)_{{h = h_{m} }}$$

4 $$h_{c} = h_{m} - \varepsilon \frac{{P_{m} }}{S}$$

where *E*_*r*_ is the indentation modulus, *E*_*i*_ means the elastic modulus of the indenter, *v*_*i*_ denotes the Poisson's ratio of the indenter. The elastic modulus and Poisson's ratio of the diamond are 1141 GPa and 0.07, respectively. *v* is the Poisson's ratio of a material and generally valued 0.2–0.3 for cement-based cement hardened paste, which was valued 0.25 in this study^23^. *S* means the contact stiffness and *h*_*c*_ the contacting depth between the indenter and sample. *A*(*h*_*c*_) represents the projected area of contact between the indenter and sample, which is related to the contact depth *h*_*c*_. *β* is a constant related to the geometry of the indenter with a value of 1.012, and $\varepsilon$ is a constant related to the shape of the indenter, which was valued 0.75.

Because of the significant difference among the concrete hydration products, the aggressiveness products of the reaction between landfill leachate and concrete are complicated, which makes the elastic moduli measured by the indentation tests are discrete. In order to obtain the distribution law of the meso-mechanical parameters of the samples, it is necessary to calculate and statistically analyze the 90 measuring points obtained from each group of experiments, so as to get the normal distribution curve and cumulative distribution curve. The normal distribution density function is

5 $$p(x) = \frac{1}{{\sqrt {2\pi s^{2} } }}exp\left[ { - \frac{{(x - \mu )^{2} }}{{2s^{2} }}} \right]$$

where *μ* is the arithmetic mean of the test value of hydration products, *s* means the standard deviation. *μ* is determined as

6 $$\mu = \frac{1}{N}\mathop \sum \limits_{k = 1}^{N} x_{k} ,s^{2} = \frac{1}{N - 1}\mathop \sum \limits_{k = 1}^{N} (x_{k} - \mu )^{2}$$

where *N* is the number of test data for each group, which is 90 in this study, and *x*_*k*_ is the elastic modulus obtained from tests.

### Uniaxial compression tests

The maximum vertical force of the uniaxial compression test apparatus used in this research can reach 1000 kN and maximum confining pressure 50 MPa. In addition, both displacement controlling mode and force controlling mode can be applied in this apparatus. Therefore, the displacement controlling mode was chosen in this study. A small axial force of preloading is applied to the sample before loading, and a loading rate of 0.002 mm/s is then kept until the specimen is damaged.

## Test results analysis

### Micron indentation test results

Past researches revealed that the main phases of concrete hydration products are CSH gel (12.6–35.3 GPa), calcium hydroxide (33.0–44.5 GPa) and ettringite (40.0–52.0 GPa)^24^. And the aggressive products of concrete due to the landfill leachate are ettringite and Friedel salt (whose elasticity modulus is similar to that of C-SH gel^25^). Based on the micron indentation tests, the distribution of the elastic modulus at different aggressived depths can be obtained, and hence the distribution of different hydration products at different corrosion sites can be effectively judged.

#### Micron indentation test on the aggressived surface

**Figure 4:**
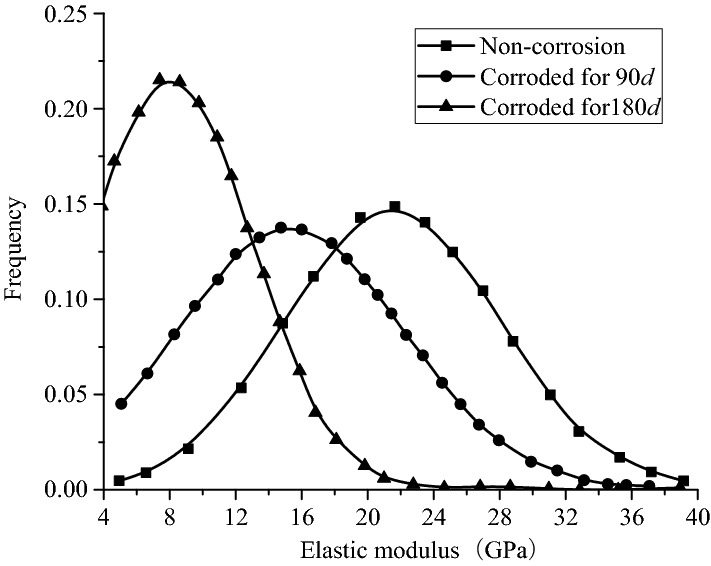
Probability fitting diagram of elastic modulus after high-concentration aggressiveness.

Table 3 shows the probability distribution of the elastic modulus of the hydration products on the cubic sample surface in corroded by group HLLN for 0, 90, and 180 days, respectively. According to Eq. (5), and the probability distribution of the test results are shown Fig. 4.

**Table 3:** Probability distribution of the elastic modulus after high-concentration aggressiveness.

Elastic modulus interval (GPa)	Corresponding probability without aggressiveness	Corresponding probability of aggressive 90d	Corresponding probability of aggressive 180d
(6,8]	0.00	0.00	0.14
(8,10]	0.00	0.07	0.27
(10,12]	0.00	0.09	0.14
(12,14]	0.04	0.17	0.10
(14,16]	0.13	0.08	0.18
(16,18]	0.13	0.17	0.10
(18,20]	0.09	0.12	0.00
(20,22]	0.16	0.07	0.02
(22,24]	0.07	0.07	0.02
(24,26]	0.07	0.02	0.02
(26,28]	0.10	0.02	0.00
(28,30]	0.02	0.04	0.00
(30,32]	0.10	0.04	0.00
(32,34]	0.04	0.02	0.00
(34,36]	0.02	0.02	0.00
(36,38]	0.00	0.00	0.00
(38,40]	0.02	0.00	0.00

Figure 4 shows that the average elastic modulus of the sample surfaces gradually decreased with aggressive time. Which also told that the average elastic modulus of hydration products was 21.55 GPa, while the average elastic modulus of hydration products on the sample surfaces decreased to 17.40 GPa and 11.23 GPa after being aggressived for 90 and 180 days, whose decrement reached 19.26% and 47.89%, respectively. Therefore, the landfill leachate has great impact on the micromechanical properties of the sample surfaces, which seriously deteriorated the micromechanical properties of the samples.

**Figure 5:**
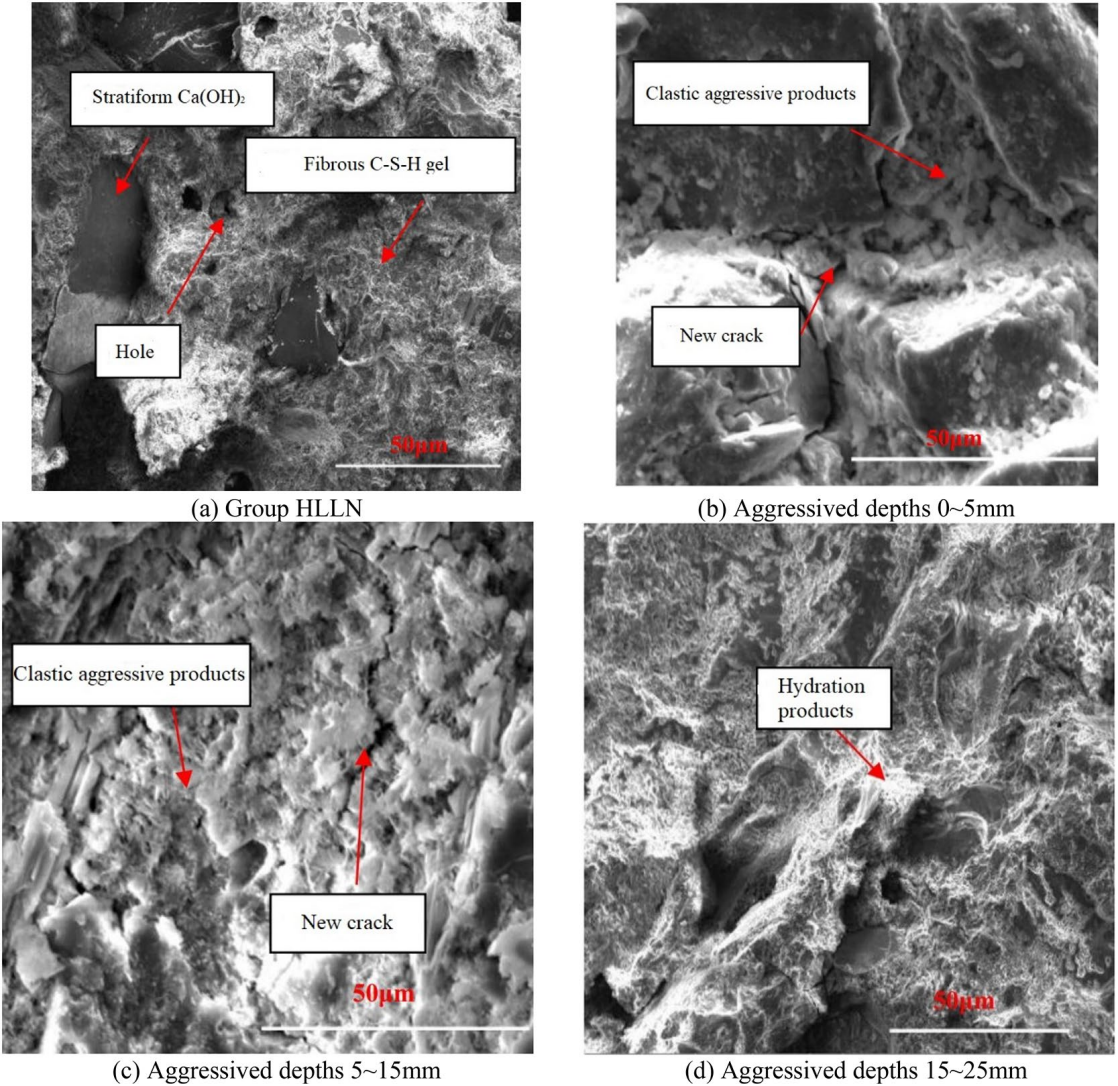
Scanning electron microscope of samples without erosion and after 180 days of erosion.

In addition, the elastic moduli of the hydration products before aggressiveness were greater than 11 GPa, most of which in the range of 27–39 GPa. It is attributed to rare pore and crack (elastic modulus in the range of 0–12.2 GPa) existed in the samples, which contained a large amount of hydration products such as C–S–H gel and calcium hydroxide with large elastic moduli (as shown in Fig. 5a). As shown in Fig. 4, with the aggressiveness continued, the distribution probability of the elastic modulus (in the range of 6–13 GPa) of the hydration products on the sample surfaces substantially increased as they were aggressived by the high-concentration landfill leachate Group HLLN for 90 days, whose probability drastically decreased with the elastic modulus in the range of 27–39 kPa. It is attributed that with the aggressiveness continued, some microscopic cracks emerged on the surface of the sample (as shown in Fig. 5b,c), which finally led to a significant increase in the probability of the elastic modulus of the hydration products in the range of 6–13 GPa. In addition, the hydration products such as C–S–H gel and calcium hydroxide in the concrete were consumed by the landfill leachate (as shown in Fig. 5d), which led to the decrease in the distribution probability of the elastic modulus in the range of 27–39 GPa. As shown in Fig. 4, after being aggressived for 180 days, the elastic modulus of the hydration products on the surface were less than 25 Gpa, which is attributed to the calcium hydroxide with high elastic modulus on the concrete surface were almost consumed by the landfill leachate, and a large number of pores, cracks. were produced. As a result, the elastic moduli in the range of 7–13 kPa of the sample surfaces were greatly increased. Therefore, the landfill leachate has great impact on the micro-mechanical properties of the concrete surfaces, which can effectively cause the overall reduction of the elastic modulus of the sample surfaces, and even lead to the deterioration of the overall micro-mechanical properties and finally result in the deterioration of the macro-mechanical properties of the samples.

#### Micro-indentation test at different aggressived depths

**Figure 6:**
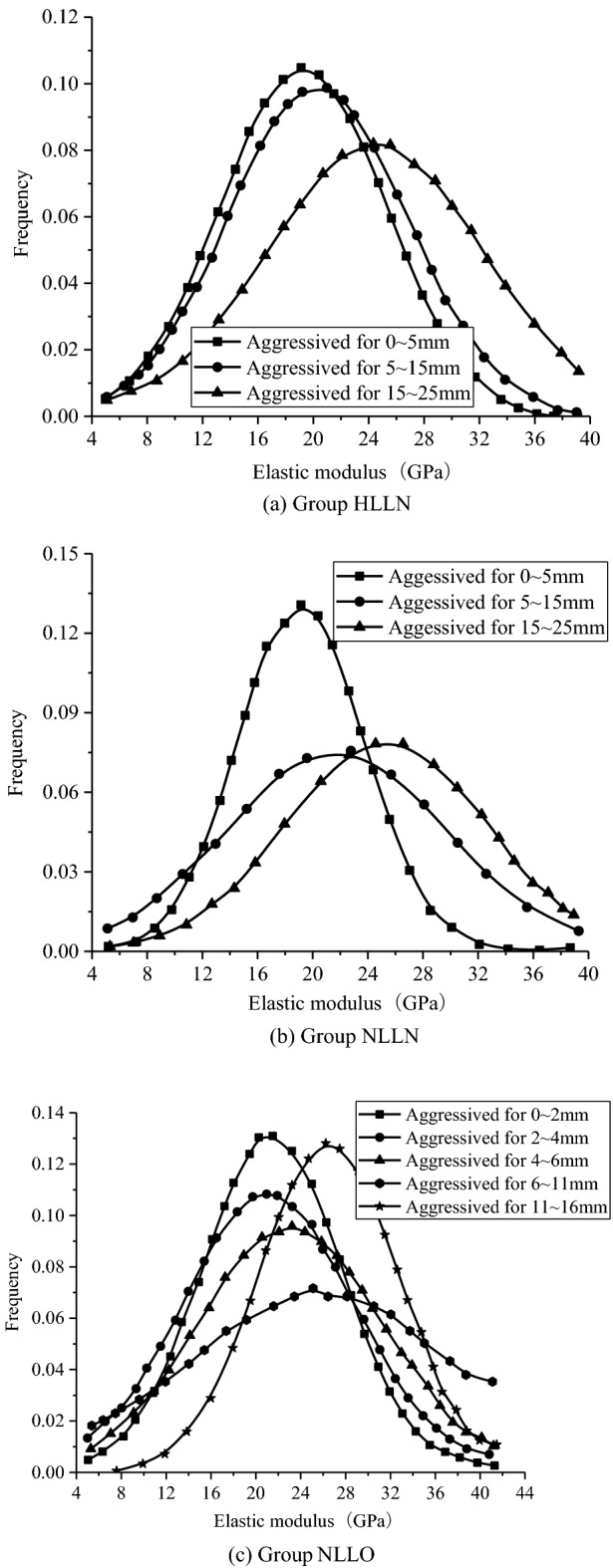
Probability fitting diagram of elastic moduli at different aggressived depths.

Local meso-mechanical properties at different aggressived depths aggressived by the high-concentration landfill leachate group HLLN, landfill leachate original solution group NLLN and landfill leachate original solution plus osmotic pressure group NLLO for 180 days were obtained through micro-indentation tests. The probability distribution of the elastic moduli of the hydration products at corrosion depth of 0–5 mm, 5–15 mm and 15–25 mm by group HLLN and group NLLN, and the elastic modulus of hydration products at samples depth 0–2, 2–4, 5–11 and 11–16 mm corroded by group NLLO are shown in Tables 4, 5 and 6, respectively. In addition, the fitting results in probability distribution diagrams are shown in Fig. 6a–c).

**Table 4:** Probability distribution of the elastic modulus at different aggressived depths for group HLLN.

Elastic modulus interval (GPa)	Corresponding probability of aggressived depth 0–5 mm	Corresponding probability of aggressived depth 5–15 mm	Corresponding probability of aggressived depth 15–25 mm
(6,8]	0.01	0.00	0.00
(8,10]	0.03	0.01	0.01
(10,12]	0.06	0.09	0.00
(12,14]	0.02	0.04	0.01
(14,16]	0.14	0.06	0.03
(16,18]	0.12	0.14	0.12
(18,20]	0.10	0.08	0.10
(20,22]	0.18	0.11	0.14
(22,24]	0.10	0.16	0.10
(24,26]	0.08	0.07	0.03
(26,28]	0.06	0.07	0.07
(28,30]	0.04	0.06	0.07
(30,32]	0.01	0.06	0.08
(32,34]	0.01	0.04	0.06
(34,36]	0.01	0.01	0.04
(36,38]	0.01	0.01	0.07
(38,40]	0.01	0.00	0.07

**Table 5:** Probability distribution of the elastic modulus at different aggressived depths for group NLLN.

Elastic modulus interval (GPa)	Corresponding probability of aggressived depth 0–5 mm	Corresponding probability of aggressived depth 5–15 mm	Corresponding probability of aggressived depth 15–25 mm
(6,8]	0.00	0.00	0.00
(8,10]	0.00	0.00	0.00
(10,12]	0.04	0.02	0.00
(12,14]	0.11	0.04	0.02
(14,16]	0.07	0.10	0.06
(16,18]	0.04	0.17	0.06
(18,20]	0.11	0.08	0.10
(20,22]	0.19	0.08	0.16
(22,24]	0.30	0.20	0.10
(24,26]	0.04	0.14	0.10
(26,28]	0.11	0.04	0.16
(28,30]	0.00	0.06	0.00
(30,32]	0.00	0.02	0.10
(32,34]	0.00	0.04	0.10
(34,36]	0.00	0.00	0.00
(36,38]	0.00	0.00	0.06
(38,40]	0.00	0.00	0.00

**Table 6:** Probability distribution of the elastic modulus at different aggressived depths for group NLLO.

Elastic modulus interval (GPa)	Corresponding probability of aggressived 0–2 mm	Corresponding probability of aggressived 2–4 mm	Corresponding probability of aggressived 4–6 mm	Corresponding probability of aggressived 6–11 mm	Corresponding probability of aggressived 11–16 mm
(8,10]	0.03	0.00	0.04	0.00	0.00
(10,12]	0.00	0.06	0.00	0.03	0.00
(12,14]	0.03	0.06	0.04	0.00	0.00
(14,16]	0.03	0.06	0.04	0.03	0.00
(16,18]	0.18	0.21	0.17	0.20	0.08
(18,20]	0.10	0.06	0.12	0.08	0.00
(20,22]	0.07	0.14	0.04	0.08	0.00
(22,24]	0.21	0.03	0.08	0.00	0.24
(24,26]	0.07	0.09	0.21	0.04	0.21
(26,28]	0.10	0.09	0.00	0.16	0.04
(28,30]	0.03	0.00	0.00	0.04	0.08
(30,32]	0.08	0.06	0.04	0.11	0.04
(32,34]	0.07	0.06	0.08	0.08	0.17
(34,36]	0.00	0.03	0.08	0.08	0.00
(36,38]	0.00	0.03	0.00	0.03	0.04
(38,40]	0.00	0.00	0.04	0.03	0.04
(40,42]	0.00	0.03	0.00	0.00	0.04

Figure 6a shows the probability distribution of the elastic modulus of hydration products at different aggressived depths before and after aggressiveness. It is obvious that the shallower the aggressived depth, the more obvious the decrease in the average elastic modulus of the hydration products. In the innermost part of the samples, due to the continued hydration of cement and secondary hydration effect of fly ash, the meso-mechanical properties were effectively enhanced. For example, the average elastic modulus of hydration products in the aggressived depth of 0–5 mm was 19.27 GPa, whose values were 20.35 GPa and 24.46 GPa in the aggressived depths of 5–15 and 15–25 mm, respectively. At the same time, the shallower the aggressived depth, the less the content of hydration products (e.g., calcium hydroxide) with larger elastic modulus, and the more aggressived products (e.g., pores and cracks) with smaller elastic modulus. Specifically, the shallower the aggressived depth, the smaller the distribution probability of the elastic modulus of hydration products in the range of 31–39 GPa, and the greater the distribution probability of the elastic modulus in the range of 5–11 GPa. On the one hand, harmful ions such as $SO_{4}^{2 - }$, $Cl^{ - }$, $HCO_{3}^{ - }$, and $H^{ + }$ in the landfill leachate reaction with the hydration products (e.g., calcium hydroxide and CSH gel), a large amount of calcium hydroxide and CSH gel were consumed and eventually led to the decrease in the distribution probability of the hydration products in the range of 31–39 GPa. On the other hand, a large number of microscopic cracks (fracture elastic modulus in the range 0–12.2 GPa) generated when the sample was aggressived, which increased the distribution probability of the elastic modulus of hydration product in the range of 5–11 GPa.

As shown in Fig. 6b, the distribution probability of the elastic modulus of the hydration product aggressived by group NLLN for 180 days are the same as those in group HLLN. Which means the shallower the aggressived depth, the smaller the average elastic modulus and the smaller the probability of the elastic modulus of hydration products in the range of 31–39 GPa, and the greater the distribution probability of the elastic modulus in the range of 5–11 GPa. Nevertheless, the average elastic modulus of hydration products was slightly larger, which is due to the aggressived degree of concrete is slightly lower.

Seeing from Fig. 6c, the distribution of the elastic modulus of the hydration products is similar to those at the aggressived depths of 0–2 mm and 2–4 mm, as well as the average elastic modulus are almost the same. With the increase in aggressived depth, the average elastic modulus of hydration products significantly increased. And at aggressived depth 11–16 mm, the average elastic modulus of hydration products reached 26.51 GPa. In addition, as the aggressived depth increased, the probability of the elastic modulus of hydration products in the range of 31–39 GPa was greater, while the distribution probability of the elastic modulus in the range of 5–11 GPa was smaller, which is in consistent with the results by the group HLLN and group NLLN.

#### Depth of sample aggressiveness

**Figure 7:**
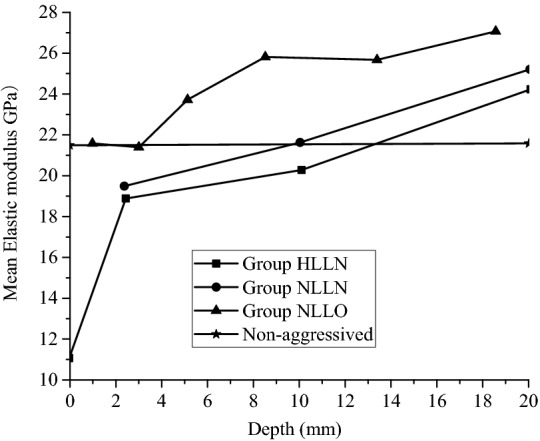
Elastic modulus at different aggressived depths.

The mechanical parameters of the surfaces and different aggressived depths can be accurately measured through micro-indentation tests. As Fig. 7 shows the relations between the mean elastic modulus of the sample surfaces and different aggressived depths versus aggressived depths without being aggressived and after being aggressived for 180 days by the high-concentration landfill leachate group HLLN, the original landfill leachate solution group NLLN, and the original landfill leachate solution plus osmotic pressure group NLLO.

Seeing from Fig. 7, the mean elastic modulus of the specimens aggressived by the three aggressive groups for 180 days significantly increase with the increase of aggressived depth, and the internal mechanical properties of the specimens were effectively enhanced. The specimens aggressived by group HLLN behaved different in the local mechanical properties at different aggressived depths. The closer the distance to the specimen surface, the more drastic change about the mechanical properties. For example, after being aggressived for 180 days, the average elastic modulus of the hydration products on the sample surface was 11.23 GPa, while the average elastic modulus of the hydration products quickly increased to 19.27 GPa at the aggressived depth of 0–5 mm, and the mean elastic modulus of the hydration products increased slowly with aggressived depth. Which indicates that the deterioration of the surface layer of the samples are far more serious than that of the inside of samples. The evolution of the mean elastic modulus of the sample aggressived by group NLLN is similar to that of group HLLN, while the mean elastic modulus of the sample aggressived by group NLLN is slightly higher than that of group HLLN at a same aggressived depth, which indicates that the sulfate ion and chloride ion in the high-concentration landfill leachate aggressive group HLLN accelerate the aggressive process. There was no significant decreasing in the elastic modulus of the samples aggressived by group NLLO, and the mean elastic modulus of the hydration products at a depth of 0–4 mm was equal to that in the unaggressived state. In addition, the mean elastic modulus of the hydration products increased with the aggressived depth. And at the same aggressived depth, it’s mean elastic modulus was greater than that of the samples aggressived by groups HLLN and NLLN. On the one hand, the osmotic pressure (0.4 MPa) applied in the test was relatively small. As a result, an effective seepage channel in the concrete did not form. On the other hand, there was a hole in the center of the sample with a diameter of 20 mm and depth of 100 mm, small osmotic pressure and dense concrete structure made it impossible for the erosive solution inside the hole to permeate and diffuse, and the aggressive speed was finally limited.

**Figure 8:**
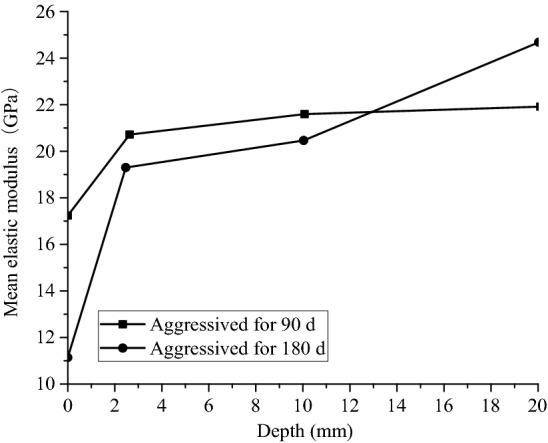
Modulus of elasticity at different aggressived depths for Group HLLN.

According to Fig. 8, when the samples are aggressived by group HLLN for 90 and 180 days, there are significant increase in the elastic moduli of the samples as the increase of aggressived depth, and the elastic moduli changed sharply at shallow aggressived depth and slowly increased at deep aggressived depth. In addition, when the aggressived depth is less than 14 mm, the mean elastic modulus of the samples aggressived for 90 days are greater than those aggressived for 180 days. If the aggressived depth is greater than 14 mm, the mean elastic moduli of the sample aggressived for 90 days are smaller than those aggressived for 180 days, which contributes to fact that the samples aggressived by the landfill leachate at the shallow aggressived depth behaved less deterioration on the micromechanical properties of the samples.

### Uniaxial compression test results

In order to study the degradation law of the macro-mechanical properties of concrete in the landfill leachate, sodium sulfate and sodium chloride solutions, the concretes were aggressived by the high-concentration landfill leachate group HLLN, sodium sulfate solution group NSSN, and sodium chloride solution group NSCN, and the uniaxial compression test results are as follows.

#### Deterioration law of compressive strength

**Figure 9:**
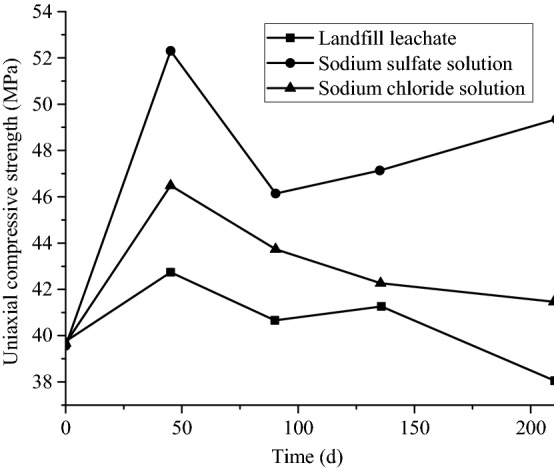
Uniaxial compressive strength of groups HLLN, NSSN, and NSCN after corrosion.

Figure 9 shows the variation of uniaxial compressive strengths of the samples after being aggressived by several solutions for a certain period of time. It is obvious that the uniaxial compressive strength can be classified into two stages: linear increase and slow decline. In the linear increase phase (from 0 to 45 days), the uniaxial compressive strength of group HLLN increased by 7.6%, whereas 31.5% and 17.3% increments took place in groups NSSN and NSCN, respectively. Hence, the uniaxial compressive strength growth of the samples in group B is significantly greater than that of in group HLLN. While in the slow decline stage (from 45 to 210 days), the uniaxial compressive strengths in groups HLLN, NSSN and NSCN showed gradual decrease trends. In addition, it is obvious that the uniaxial compressive strengths corresponding to group NSSN are highest, and the strengths corresponding to group HLLN are lowest in the above two stages. After being aggressived for 210 days, the uniaxial compressive strengths corresponding to group NSSN and NSCN respectively increased by 20% and 5%, while the strength corresponding to group HLLN decreased by 5%. Therefore, it is concluded that compared with the sodium chloride and sodium sulfate solutions, the impact of landfill leachate on the strength of concrete and its macro-mechanical properties are the most significant in the short term.

According to Fig. 9, the uniaxial compressive strengths of the three conditions in the first stage show different increasing degrees, which attributes to the continuous the hydration of concrete and secondary hydration effect of fly ash. In the linear increasing stage, because the components in landfill leachate of group HLLN weakened the above-mentioned effects, which eventually resulted in the lowest increase in the uniaxial compressive strength. While the compressive strength corresponding to group NSSN experienced a process of first decreasing and then increasing, which is due to the divalent sulfate ion reaction with the ions in the concrete, and the material produced by the reaction gradually hardened afterwards. After that, the compressive strength of group NSCN experienced a gradual decrease, the possible reason is that the aggressiveness effect of chloride ions on the concrete is gradually increased from the surface to inside. Therefore, the subsequent compressive strength change trend of group HLLN lies between group NSSN trend and group NSCN. In other words, it experienced a change from first decline, then a slight increase, and finally decline.

#### Deterioration law of elastic modulus

**Figure 10:**
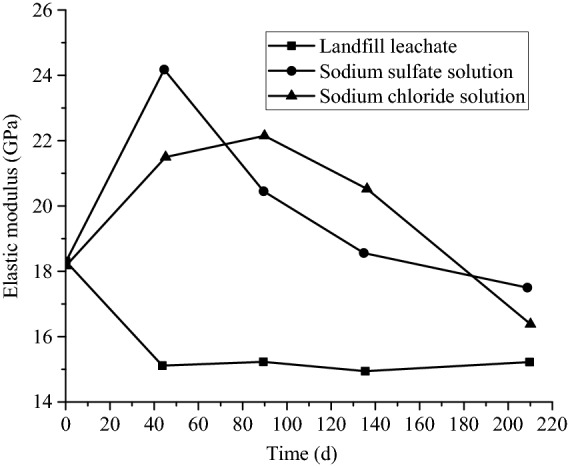
Elastic modulus of groups HLLN, NSSN, and NSCN after aggressiveness.

According to^26^, the trends of elastic modulus for the three groups are shown in Fig. 10. It is obvious that the elastic moduli of the samples in landfill leachate decreased by 17.4% after being aggressived by group HLLN for 45 days, whose modulus changed from 18.274 to 15.092 GPa, and finally kept relatively stable. While the elastic modulus of the samples aggressived by group NSSN in the sodium sulfate solution increased by 32.2% corroded for 45 days, which finally reached 24.16 GPa and finally gradually decreased. Nevertheless, the elastic modulus of the group NSCN in the sodium chloride solution showed a relatively stable growth firstly and then gradually decreased. After being aggressived by the three solutions for 210 days, the elastic modulus of group HLLN, NSSN and NSCN decreased by 16.1%, 2.8% and 10.4%, respectively. Therefore, it is concluded that compared with the sodium sulfate and sodium chloride solutions, the change in the elastic modulus of concrete is more obvious after aggressived by the landfill leachate.

## Conclusion

In order to study the evolution of the macro/micromechanical properties of the concrete aggressived by landfill leachate, this research focused on the behavior of the surfaces and different aggressived depths aggressived by high-concentration landfill leachate group HLLN, sodium sulfate solution group NSSN, sodium chloride solution group NSCN, landfill leachate original solution group NLLN and landfill leachate original solution plus osmotic pressure group NLLO. The main conclusions are as follows:

1. As the aggressiveness of the landfill leachate continues, a large number of microscopic cracks generated on the surface of the samples. At the same time, the hydration products with high elastic modulus such as C–S–H gel and calcium hydroxide in the concrete were consumed by the landfill leachate. The longer the aggressiveness of the high-concentration landfill leachate, the more severe the deterioration of the micromechanical properties of the sample surface.
2. The shallower the aggressived depth, the more severe the deterioration of the local meso-mechanical properties of the samples, the more severe the change in the mechanical properties, the less the content of hydration products with larger elastic modulus such as calcium hydroxide, and the more aggressive products with smaller elastic modulus such as pore and cracks.
3. The uniaxial compressive strength and elastic modulus of the concrete samples aggressived by landfill leachate showed a linear increase first and then slowly decrease with time. Compared with the sodium chloride and sodium sulfate solutions, the landfill leachate has the most significant weakening effect on the elastic modulus of concrete.
